# Two new species of oribatid mites of *Lasiobelba* (Acari, Oribatida, Oppiidae) from Nepal, including a key to all species of the genus

**DOI:** 10.3897/zookeys.424.7990

**Published:** 2014-07-08

**Authors:** Sergey G. Ermilov, Umukusum Ya. Shtanchaeva, Luis S. Subías, Jochen Martens

**Affiliations:** 1 Tyumen State University, Tyumen, Russia; 2 Complutense University, Madrid, Spain; 3 Johannes Gutenberg University, Mainz, Germany

**Keywords:** Oribatid mites, new species, *Lasiobelba*, key, Nepal

## Abstract

Two new species of oribatid mites of the genus *Lasiobelba* (Oribatida, Oppiidae), *Lasiobelba* (*Lasiobelba*) *daamsae* **sp. n.** and *Lasiobelba* (*Antennoppia*) *nepalica* **sp. n.**, are described from eastern Nepal. *Lasiobelba* (*L.*) *daamsae* **sp. n.** is most similar to *L.* (*L.*) *remota* Aoki, 1959 and *L.* (*L.*) *gibbosa* (Mahunka, 1985), however, it differs from both by the anterior part of pedotecta I specifically curved, rostrum pointed and exobothridial setae not shorter than bothridial setae. *Lasiobelba* (*Antennoppia*) *nepalica* **sp. n.** is most similar to *L.* (*A.*) *granulata* (Mahunka, 1986), however, it differs from the latter by the larger body size, exobothridial setae longer than rostral setae and bothridial setae not longer than interlamellar setae. An identification key to known species of *Lasiobelba* is given.

## Introduction

*Lasiobelba* is a genus of oribatid mites (Oribatida, Oppiidae, Oppiinae) that was proposed by Aoki (1959) with *Lasiobelba remota* Aoki, 1959 as type species. The main generic characters (summarized by Aoki 1959; Subías and Balogh 1989; Ohkubo 2001; including our additions) are: costulae and transcostula absent; prodorsal setae well developed, setiform (exception: interlamellar setae represented by alveoli); bothridial setae spindle-form or setiform; notogaster with nine to 10 pairs of notogastral setae (setae *c* reduced, minute or represented by alveoli); dorsal notogastral setae inserted in four subparallel rows, rarely in two parallel rows; genital plates with five pairs of genital setae; adanal lyrifissures located near to anal aperture.

Currently, *Lasiobelba* comprises two subgenera (*Lasiobelba (Lasiobelba)* Aoki, 1959, *Lasiobelba (Antennoppia)* Mahunka, 1983 – see Mahunka 1983a) and 32 species, which have a cosmopolitan distribution (Subías 2004, updated 2014). The subgenus *Lasiobelba (Lasiobelba)* differs from *Lasiobelba (Antennoppia)* by the morphology of bothridial setae (spindle-form versus setiform).

In the course of taxonomic identification of oribatid mites from Nepal^1^ (Ermilov et al. 2013, 2014; Ermilov and Martens 2014), we found two new species of the genus *Lasiobelba*; one belonging to *Lasiobelba (Lasiobelba)*, other to *Lasiobelba (Antennoppia)*. The first goal of our paper is to describe these species. The second goal of our paper is to present an identification key to all known species of *Lasiobelba*.

## Materials and methods

Five specimens (holotype: male; four paratypes: all males) of *Lasiobelba (Lasiobelba) daamsae* sp. n. are from: eastern Nepal, 27°19'N, 87°78'E, Panchthar District, upper course of Mai Majuwa river, pasture Dhorpar Kharka, soil in mixed broadleaved forest, 2770 m a.s.l., 27–28.VIII.1983, collected by J. Martens and B. Daams. Four specimens (holotype: male; three paratypes: two males and one female) of *Lasiobelba (Antennoppia) nepalica* sp. n. are from: eastern Nepal, 26°99'N, 86°67'E, Ilam District, soil in remnants of broadleaved forest with plantations of *Cryptomeria japonica*, 2100 m a.s.l., 31.III.–01.IV.1980, collected by J. Martens and A. Ausobsky.

Holotypes and paratypes were mounted in lactic acid on temporary cavity slides for measurement and illustration. The body length was measured in lateral view, from the tip of the rostrum to the posterior edge of the ventral plate. The notogastral width refers to the maximum width in dorsal aspect. Lengths of body setae were measured in lateral aspect. All body measurements are presented in micrometers. Formula for leg setation is given in parentheses according to the sequence trochanter–femur–genu–tibia–tarsus (famulus included). Formula for leg solenidia is given in square brackets according to the sequence genu–tibia–tarsus. General terminology used in this paper follows that of Norton and Behan-Pelletier (2009).

## Taxonomy

### Description of Lasiobelba (Lasiobelba) daamsae sp. n.

Taxon classificationAnimaliaOribatidaOppiidae

http://zoobank.org/EE1BC06A-8B49-4004-B3EA-D1FF46336897

Figs 1 – 9

#### Diagnosis.

Body size: 1278–1310 × 747–863. Rostrum pointed. Prodorsal setae long, barbed; *in* ≈ *le* > *ss* ≈ *ex* > *ro.* Bothridial setae spindle-form, with long, thin apex, barbed. Nine pairs of notogastral setae long, barbed (*p*_1_–*p*_3_ shorter than others). Antero-medial part of rutelli with tooth. Anterior part of pedotecta I specifically curved. Anogenital setae barbed. Dorsal side of leg claws with small teeth.

#### Description.

*Measurements*. Body length: 1294 (holotype, male), 1278–1310 (four paratypes: males); notogaster width: 796 (holotype), 747–863 (four paratypes).

**Figure 1.**
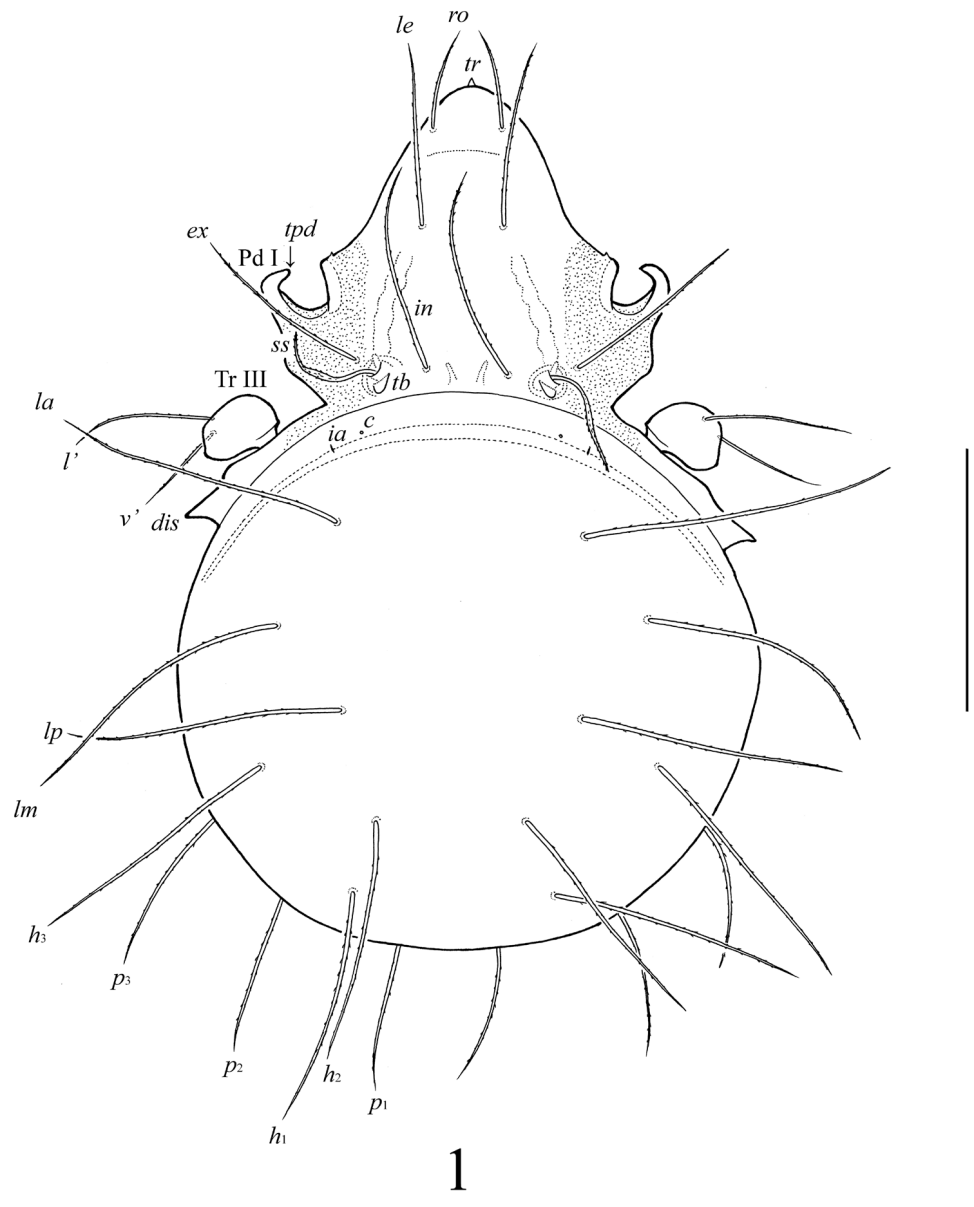
*Lasiobelba (Lasiobelba) daamsae* sp. n.: dorsal view (legs except trochanters III not illustrated). Scale bar 400 μm.

*Integument* (Figs 1, 3). Body color light brownish. Body surface smooth, but lateral parts of prodorsum with microgranulate cerotegument (diameter granules less than 1).

**Figure 2.**
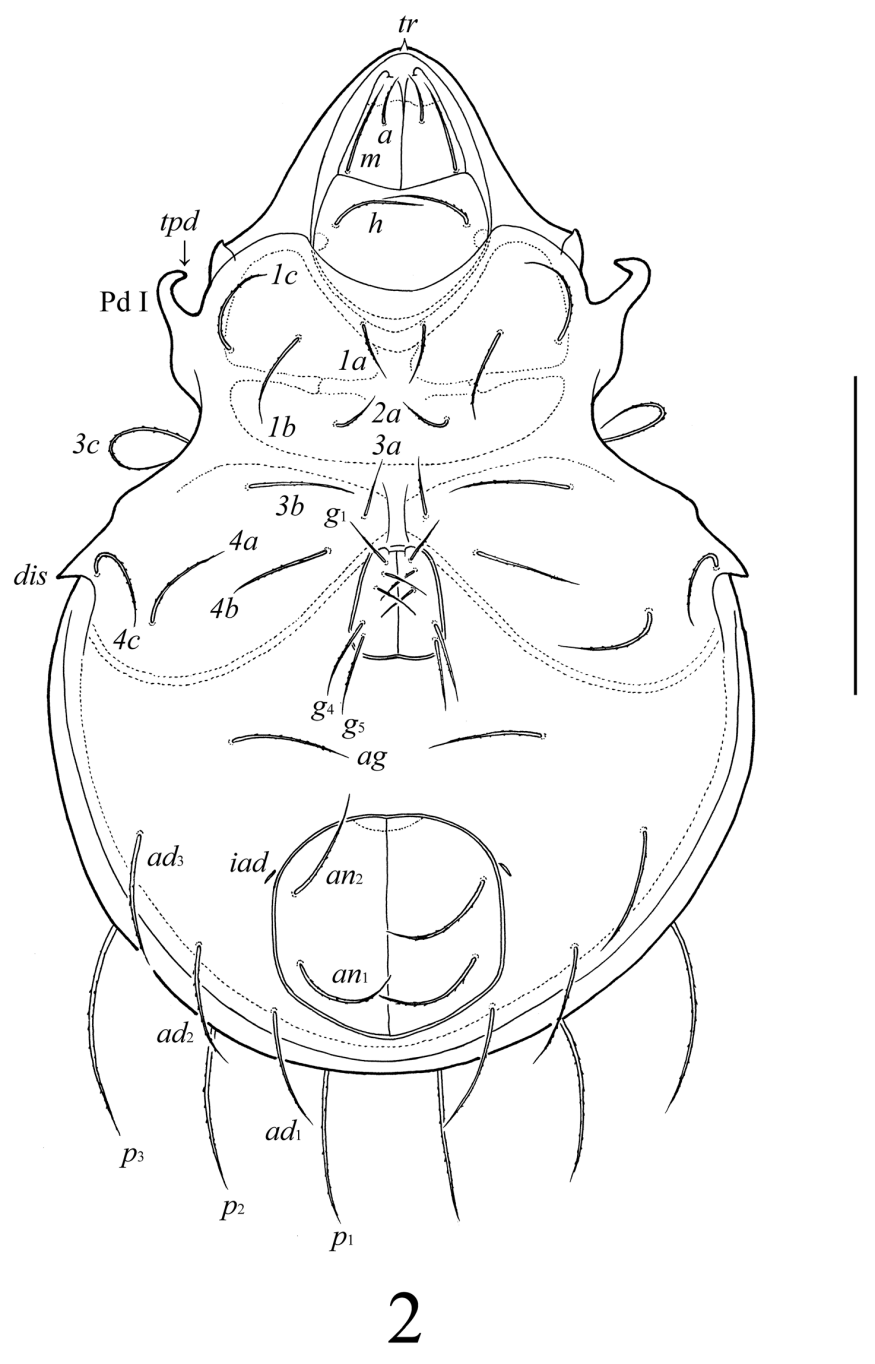
*Lasiobelba (Lasiobelba) daamsae* sp. n.: ventral view (legs not illustrated). Scale bar 400 μm.

**Figures 3–9.**
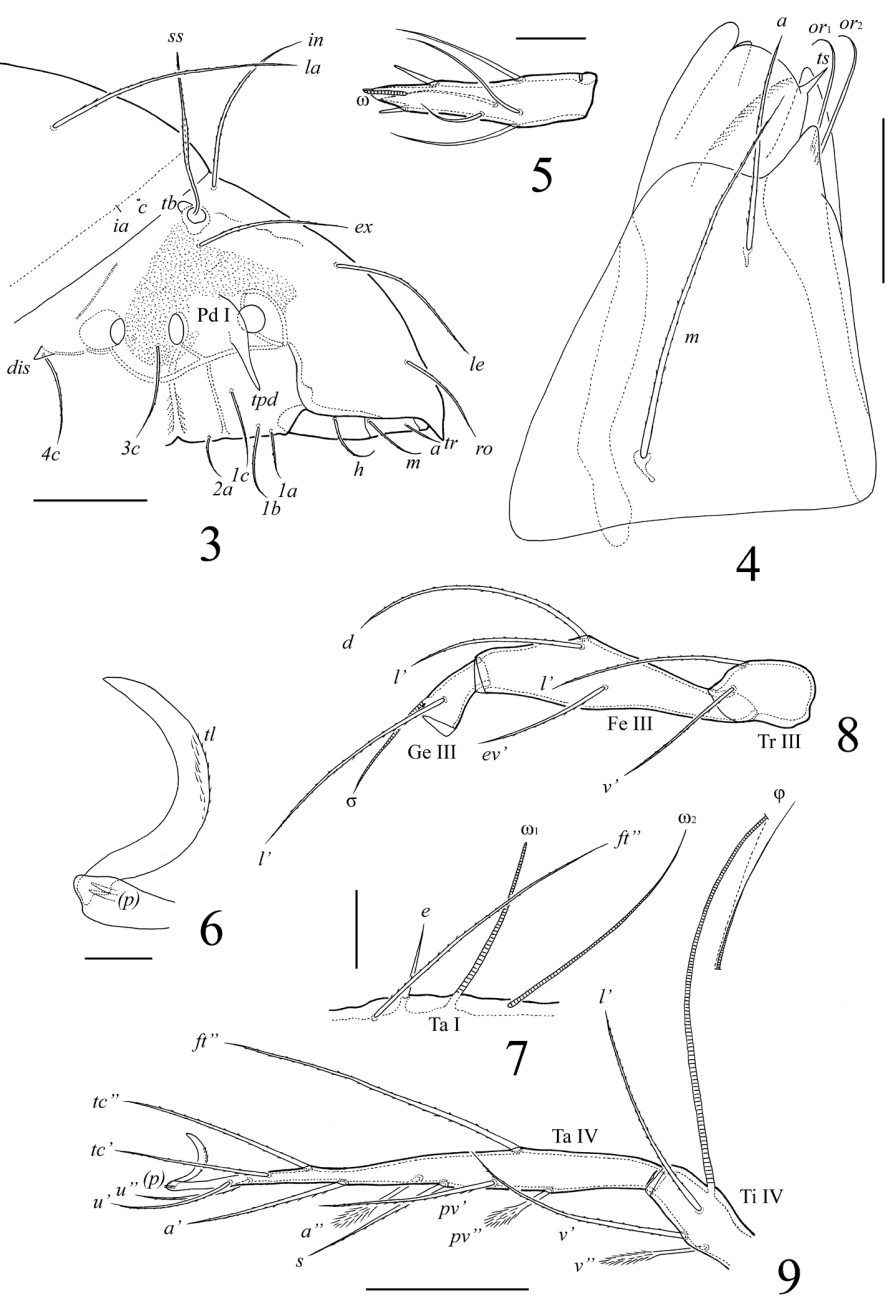
*Lasiobelba (Lasiobelba) daamsae* sp. n.: **3** lateral view of prodorsum (legs not illustrated) and anterior part of notogaster **4** right rutellum and gena of subcapitulum, ventral view **5** palptarsus **6** leg claw II and setae *p* **7** localization of solenidia, famulus and seta *ft*’’ on tarsus I, right, antiaxial view **8** trochanter, femur and genu of leg III, right, antiaxial view **9** tarsus and anterior part of tibia of leg IV, right, antiaxial view. Scale bars 200 μm (**3, 8, 9**), 50 μm (**4, 7**), 20 μm (**5, 6**).

*Prodorsum* (Figs 1–3). Rostrum with conical tooth (*tr*, 12–16). A row, comprising several muscle sigillae, is located in front of the bothridia (usually very poorly visible). Muscle sigilla in interbothridial region absent, but one pair of longitudinal, dark brown structures are present. Rostral (*ro*, 199–232), lamellar (*le*, 365–381), interlamellar (*in*, 365–381) and exobothridial (*ex*, 265–298) setae well developed, setiform, barbed. Bothridial setae (*ss*, 265–298) spindle-form, barbed, with weakly developed elongate head and long, thin apex. A pair of triangular tubercles (*tb*) located posteriorly to bothridia.

*Notogaster* (Figs 1–3). Anterior border convex. Notogastral setae *c* represented by alveolus. Nine other pairs of notogastral setae long, barbed; *p*_1_–*p*_3_ (215–249) shorter than others (431–481). Lyrifissures *ia* poorly visible, *im*, *ip*, *ih*, *ips* and opisthonotal gland openings present, but visible under high magnification in dissected specimens.

*Gnathosoma* (Figs 2, 4, 5). Subcapitulum longer than wide (298–315 × 199–215). Antero-medial part of rutelli with tooth (*ts*, 8). Subcapitular setae setiform, barbed; *a* (66–83) shorter than *m* and *h* (both 116–132). Two pairs of adoral setae (*or*_1_, *or*_2_, 33–49) setiform, hook-like distally, smooth. Palps (199) with setation 0–2–1–3–8(+ω). Solenidion thickened, blunt-ended, pressed to the palptarsus surface in basal part and distal seta in distal part. Chelicerae (298–315) with two barbed setae; *cha* (99) longer than *chb* (66). One short tooth (4–6) located posteriorly to seta *cha.* Trägårdh’s organ distinct.

*Epimeral and lateral podosomal regions* (Figs 1–3). Apodemes (1, 2, sejugal, 4) weakly developed. Epimeral setae setiform, barbed; setae *1a*, *2a*, *3a* (83–99) shorter than *1b*, *1c*, *3b*, *4a*, *4b* (149–166) and *3c*, *4c* (199–232). Anterior part of pedotecta I (Pd I) elongate and specifically curved, forming a tooth (*tpd*). Discidia (*dis*) triangular, pointed.

*Anogenital region* (Figs 2, 3). Five pairs of genital (*g*_1_–*g*_3_, 74–83; *g*_4_, *g*_5_, 108–116), one pair of aggenital (*ag*, 166–199), three pairs of adanal (*ad*_1_–*ad*_3_, 166–199) and two pairs of anal (*an*_1_, *an*_2_, 149–166) setae setiform, barbed. Distance between setae *ad*_3_–*ad*_3_ longer than *ad*_2_–*ad*_2_ and *ad*_1_–*ad*_1_. Adanal lyrifissures *iad* located diagonally, but very close to anal aperture.

*Legs* (Figs 1, 6–9). Generally, morphology typical for species of *Lasiobelba* (Bernini 1973; Ohkubo 2001; Ermilov and Kalúz 2012). Dorsal side of each claw in all tarsi with two rows of small teeth (*tl*). Formulae of leg setation and solenidia: I (1–5–2–4–20) [1–2–2], II (1–5–2–4–16) [1–1–2], III (2–3–1–3–15) [1–1–0], IV (1–2–2–3–12) [0–1–0]; homology of setae and solenidia indicated in Table 1. Setae *p* setiform on tarsi I, very short, conical on tarsi II–IV. Famulus (*ε*) setiform, straight, pointed, inserted posteriorly to solenidion ω_1_.

**Table 1.** Leg setation and solenidia of *Lasiobelba (Lasiobelba) daamsae* sp. n. (same data for *Lasiobelba (Antennoppia) nepalica* sp. n.).

Leg	Trochanter	Femur	Genu	Tibia	Tarsus
I	*v*’	*d*, *(l)*, *bv*’’, *v*’’	*(l)*, σ	*(l)*, *(v)*, φ_1_, φ_2_	*(ft)*, *(tc)*, *(it)*, *(p)*, *(u)*, *(a)*, *s*, *(pv)*, *v*’, *(pl)*, *l*’’, *ε*, *ω*_1_, *ω*_2_
II	*v*’	*d*, *(l)*, *bv*’’, *v*’’	*(l)*, σ	*(l)*, *(v)*, φ	*(ft)*, *(tc)*, *(it)*, *(p)*, *(u)*, *(a)*, *s*, *(pv)*, *l*’’, *ω*_1_, *ω*_2_
III	*l*’, *v*’	*d*, *l*’, *ev*’	*l*’, σ	*l*’, *(v)*, φ	*(ft)*, *(tc)*, *(it)*, *(p)*, *(u)*, *(a)*, *s*, *(pv)*
IV	*v*’	*d*, *ev*’	*d*, *l*’	*l*’, *(v)*, φ	*ft*’’, *(tc)*, *(p)*, *(u)*, *(a)*, *s*, *(pv)*

Roman letters refer to normal setae (*ε* to famulus), Greek letters to solenidia. Single prime (’) marks setae on anterior and double prime (’’) setae on posterior side of the given leg segment. Parentheses refer to a pair of setae.

#### Type deposition.

The holotype and one paratype are deposited in the collection of the Senckenberg Institution Frankfurt, Germany; three paratypes are deposited in the collection of the Tyumen State University Museum of Zoology, Tyumen, Russia.

#### Etymology.

The specific name is dedicated to Mrs. Beate Daams for her assistance in Nepalese scientific researches.

#### Remarks.

In having the long notogastral setae, large body size and spindle-form bothridial setae, *Lasiobelba (Lasiobelba) daamsae* sp. n. is most similar to *Lasiobelba (Lasiobelba) remota* Aoki, 1959 from the Oriental and Palaearctic regions and *Lasiobelba (Lasiobelba) gibbosa* (Mahunka, 1985) from the Ethiopian region. However, it differs from both by the anterior part of pedotecta I specifically curved (versus straight in *Lasiobelba (Lasiobelba) remota* and *Lasiobelba (Lasiobelba) gibbosa*), rostrum pointed (versus rounded in *Lasiobelba (Lasiobelba) remota* and nasiform in *Lasiobelba (Lasiobelba) gibbosa*) and exobothridial setae not shorter than bothridial setae (versus shorter in *Lasiobelba (Lasiobelba) remota* and *Lasiobelba (Lasiobelba) gibbosa*).

### Description of Lasiobelba (Antennoppia) nepalica sp. n.

Taxon classificationAnimaliaOribatidaOppiidae

http://zoobank.org/F023D27B-A28D-4A1F-B987-3834F4DF4E97

Figs 10 – 15

#### Diagnosis.

Body size: 996–1278 × 697–830. Prodorsal setae long, barbed; *ss* ≈ *in* > *le* > *ex* > *ro.* Nine pairs of notogastral setae long, barbed (*p*_1_–*p*_3_ shorter than others). Antero-medial part of rutelli with tooth. Anogenital setae barbed. Dorsal side of leg claws with small teeth.

#### Description.

*Measurements*. Body length: 1195 (holotype, male), 996–1278 (three paratypes: two males and one female); notogaster width: 730 (holotype), 697–830 (three paratypes).

**Figure 10.**
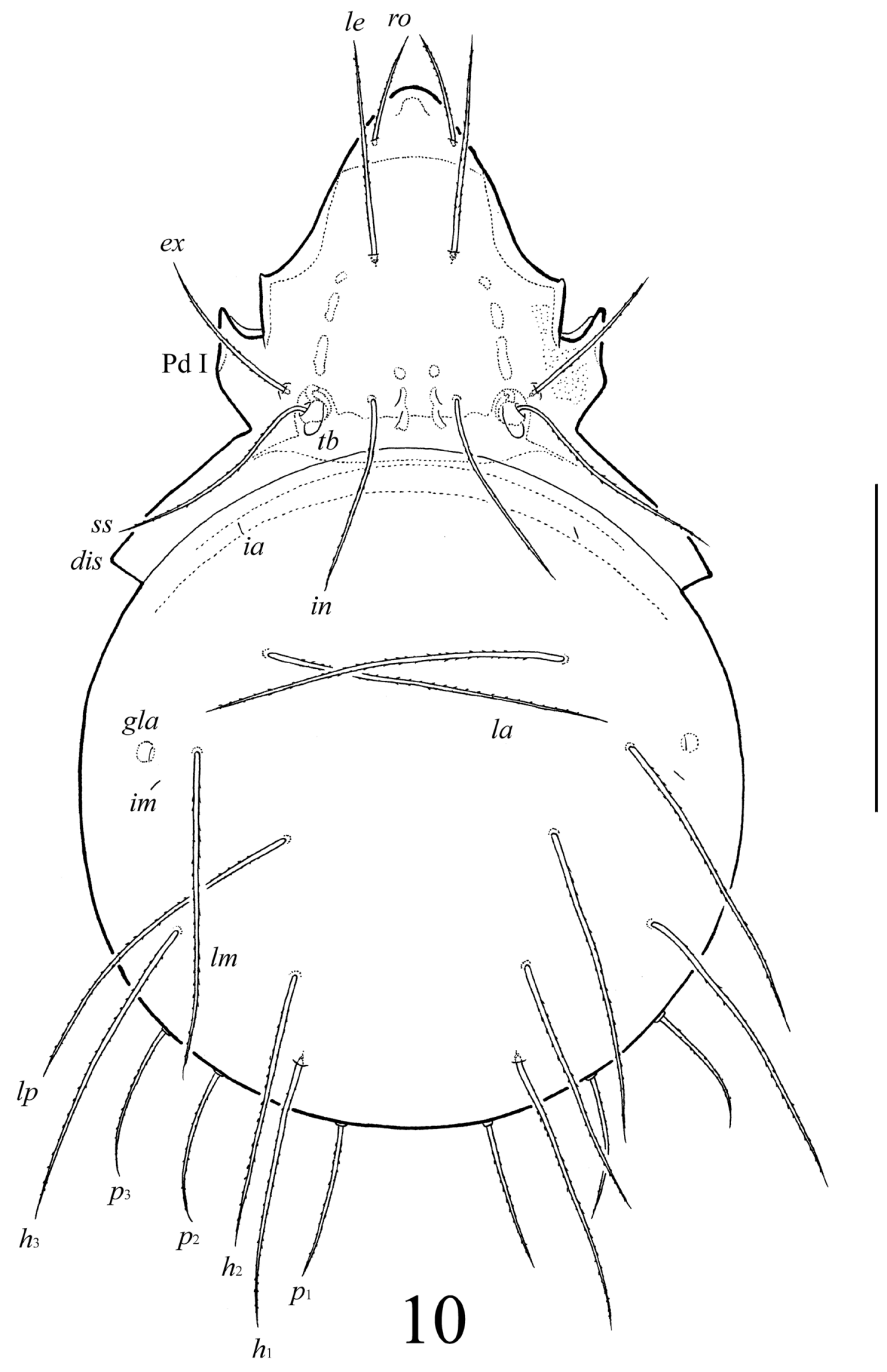
*Lasiobelba (Antennoppia) nepalica* sp. n.: dorsal view (legs not illustrated). Scale bar 400 μm.

*Integument* (Figs 10, 12). Body color light brownish. Body surface smooth, but lateral parts of prodorsum with microgranulate cerotegument (diameter granules up to 1).

*Prodorsum* (Figs 10, 12). Rostrum widely or narrowly rounded. A row, comprising several muscle sigillae, is located in front of the bothridia. One pair of muscle sigilla in interbothridial region poorly visible. Rostral (143–164), lamellar (254–287), interlamellar (307–348), exobothridial (205–258) and bothridial (307–348) setae well developed, setiform, barbed. A pair of triangular tubercles located posteriorly to bothridia.

**Figure 11.**
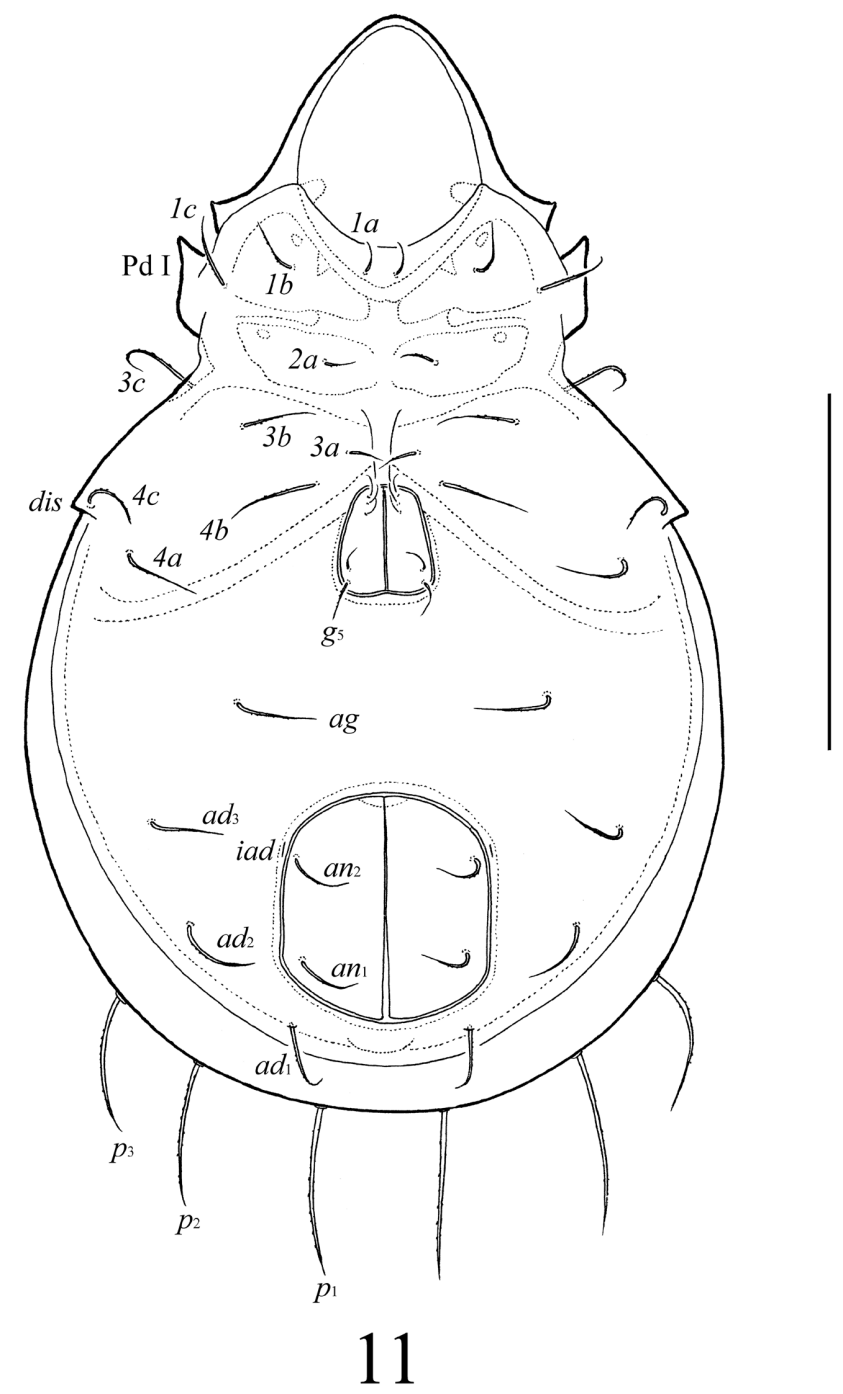
*Lasiobelba (Antennoppia) nepalica* sp. n.: ventral view (gnathosoma and legs not illustrated). Scale bar 400 μm.

**Figures 12–15.**
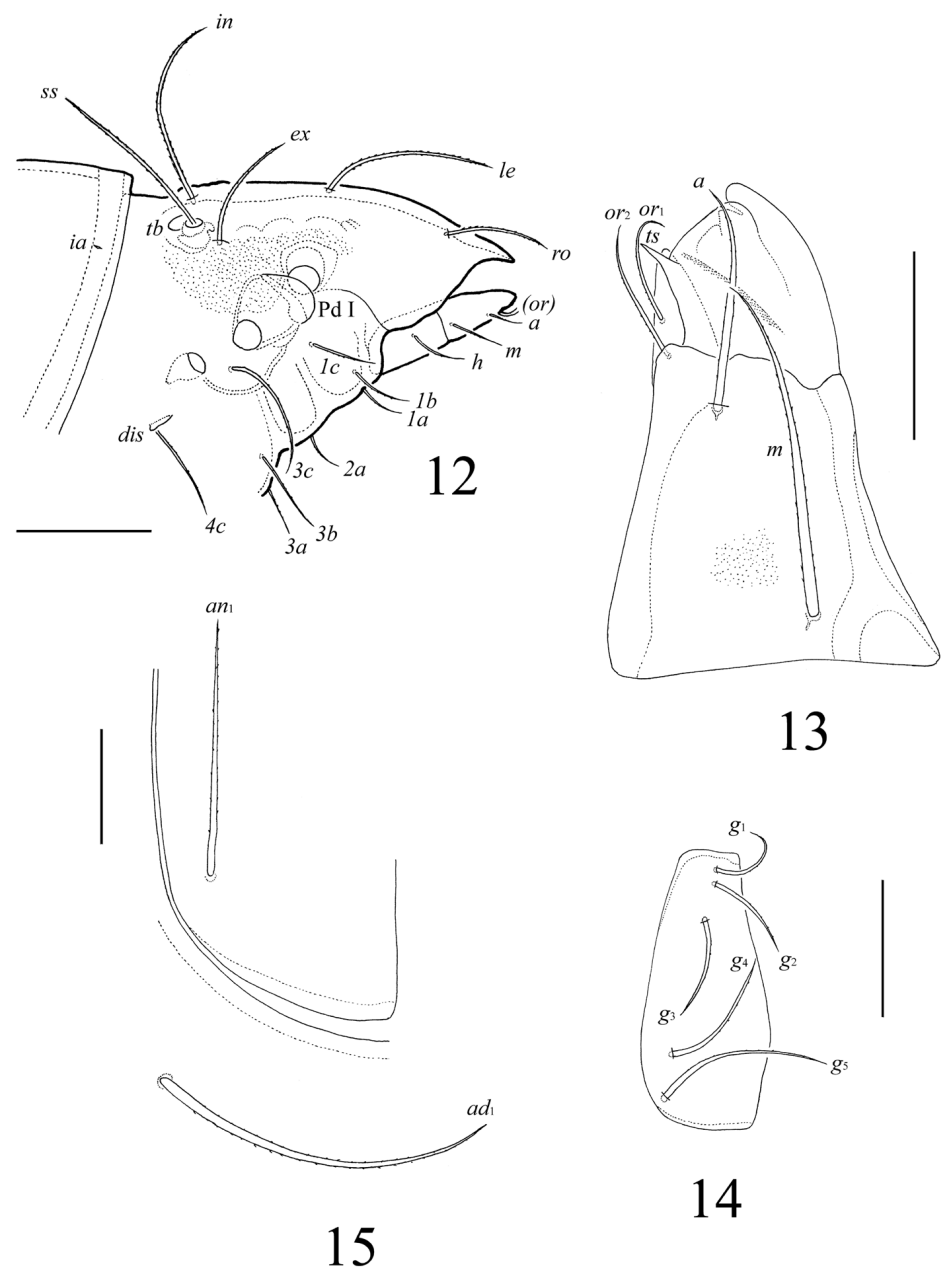
*Lasiobelba (Antennoppia) nepalica* sp. n.: **12** lateral view of prodorsum (legs not illustrated) and anterior part of notogaster **13** left rutellum and gena of subcapitulum, ventral view **14** genital plate, right **15** posterior part of anal plate with seta *an*_1_ and adanal seta *ad*_1_. Scale bar 50 μm.

*Notogaster* (Figs 10–12). Anterior border convex. Notogastral setae *c* and their alveoli reduced. Nine pairs of notogastral setae long, barbed; *p*_1_–*p*_3_ (184–192) shorter than *h*_1_, *h*_2_ (265–332) and others (398–464). Lyrifissures *ia*, *im* and opisthonotal gland openings (*gla*) poorly visible; lyrifissures *ip*, *ih*, *ips* present, but visible under high magnification in dissected specimens.

*Gnathosoma* (Figs 12, 13). Subcapitulum longer than wide (266 × 199–209). Antero-medial part of rutelli with tooth (8–10). Subcapitular setae setiform, barbed; *a* (61–65) shorter than *m* and *h* (both 98–102). Two pairs of adoral setae (41–45) setiform, indistinctly smooth. Palps (196) with setation 0–2–1–3–8(+ω). Solenidion thickened, blunt-ended, pressed to the palptarsus surface in basal part and distal seta in distal part. Chelicerae (266) with two barbed setae; *cha* (86) longer than *chb* (53). One short tooth (4–6) located posteriorly to seta *cha.* Trägårdh’s organ distinct.

*Epimeral and lateral podosomal regions* (Figs 10–12). Apodemes (1, 2, sejugal, 4) weakly developed. Epimeral setae setiform, barbed; setae *1a*, *2a*, *3a* (69–86) shorter than *1b*, *1c*, *3b*, *4a*, *4b* (114–127), *3c* (205–209) and *4c* (155–164). Pedotecta I normally developed, scale-like. Discidia triangular, pointed.

*Anogenital region* (Figs 11, 14, 15). Five pairs of genital setae (*g*_1_–*g*_3_, 41–53; *g*_4_, 61–69, *g*_5_, 73–82) setiform, indistinctly barbed. One pair of aggenital (123–135), three pairs of adanal (159–172) and two pairs of anal (114–123) setae setiform, barbed. Distance between setae *ad*_3_–*ad*_3_ longer than *ad*_2_–*ad*_2_ and *ad*_1_–*ad*_1_. Adanal lyrifissures *iad* located longitudinally.

*Legs*. Generally, similar to *Lasiobelba (Lasiobelba) daamsae* sp. n. (see also Table 1).

#### Type deposition.

The holotype and one paratype are deposited in the collection of the Senckenberg Institution Frankfurt, Germany; two paratypes are deposited in the collection of the Tyumen State University Museum of Zoology, Tyumen, Russia.

#### Etymology.

The specific name “*nepalica*” refers to the country origin, Nepal.

#### Remarks.

In having the long prodorsal and notogastral setae and large body size, *Lasiobelba (Antennoppia) nepalica* sp. n. is most similar to *Lasiobelba (Antennoppia) granulata* (Mahunka, 1986) from Tanzania. However, it clearly differs from the latter by the larger body size (996–1278 × 697–830 versus 820–861 × 541–574 in *Lasiobelba (Antennoppia) granulata*), exobothridial setae longer than rostral setae (versus rostral longer in *Lasiobelba (Antennoppia) granulata*) and bothridial setae not longer than interlamellar setae (versus clearly longer in *Lasiobelba (Antennoppia) granulata*).

### Key to known species of *Lasiobelba*^2^

**Table:** 

1	Bothridial setae spindle-form	**2, subgenus *Lasiobelba (Lasiobelba)***
–	Bothridial setae setiform	**18, subgenus *Lasiobelba (Antennoppia)***
2	Dorsal notogastral setae long, *lm* reaching the insertions of *lp*	**3**
–	Dorsal notogastral setae of medium size or short, *lm* not reaching the insertions of *lp*	**13**
3	Notogastral setae *la*, *lm*, *lp* longer than bothridial setae	**4**
–	Notogastral setae *la*, *lm*, *lp* shorter than bothridial setae	**10**
4	Rostrum pointed	**5**
–	Rostrum widely or narrowly rounded, or truncated	**6**
5	Anterior part of pedotecta I specifically curved; notogastral setae *p*_1_–*p*_3_ longer than adanal setae; body size: 1278–1310 × 747–863	***Lasiobelba (Lasiobelba) daamsae* sp. n.** Distribution: Nepal
–	Pedotecta I normally developed; notogastral setae *p*_1_–*p*_3_ shorter than adanal setae; body size: 772–891 × 410–456	***Lasiobelba (Lasiobelba) gibbosa* (Mahunka, 1985)**. Distribution: Ethiopian region
6	Interlamellar setae similar in length (little longer or shorter) to bothridial setae	**7**
–	Interlamellar setae clearly shorter than bothridial setae	**9**
7	Rostrum truncated; body size: 794–834 × 492–564	***Lasiobelba (Lasiobelba) insulata* Ohkubo, 2001**. Distribution: Japan
–	Rostrum widely or narrowly rounded	**8**
8	Rostrum widely rounded; notogastral setae *p*_1_–*p*_3_ inserted close to each other; body size: 560 × 330	***Lasiobelba (Lasiobelba) subuligera* (Berlese, 1916)** (see also Mahunka and Mahunka-Papp 1995). Distribution: Argentina
–	Rostrum with protruding ledge; notogastral setae *p*_1_–*p*_3_ clearly distanced from each other; body size: 940–1030 × 620–650	***Lasiobelba (Lasiobelba) remota* Aoki, 1959**. Distribution: Palaearctic and Oriental regions
9	Bothridial setae with head without long apex; interbothridial region with two pairs of muscle sigilla; body size: 950 × 630	***Lasiobelba (Lasiobelba) suchetae* Sanyal, 1992**. Distribution: India
–	Bothridial setae with long, thin apex; interbothridial region without muscle sigilla; body size: 625–684 × 388–437	***Lasiobelba (Lasiobelba) vietnamica* Balogh, 1983** (see Balogh and Mahunka 1967). Distribution: Vietnam
10	Notogastral setae *c* short, present	**11**
–	Notogastral setae *c* represented by alveoli	**12**
11	Anterior part of notogaster smooth; epimeral setae *1а*, *2а*, *3а* thin, almost smooth; body size: 478–522 × 277–315	***Lasiobelba (Lasiobelba) lemurica* Mahunka, 1997**. Distribution: Madagascar
–	Anterior part of notogaster microfoveolate; epimeral setae *1а*, *2а*, *3а* heavily barbed; body size: 566 × 307	***Lasiobelba (Lasiobelba) pontica* Vasiliu & Ivan, 2011**. Distribution: Romania
12	Body surface of notogaster with longitudinal ridges; interbothridial region with one pair of tubercles; body size: 693 × 455	***Lasiobelba (Lasiobelba) sculptra* Wang, 1993**. Distribution: southern China
–	Body surface of notogaster granulate; interbothridial region without tubercles; body size: 610–644 × 386–402	***Lasiobelba (Lasiobelba) yunanensis* Wen, 1999**. Distribution: southern China
13	Notogastral setae *c* represented by alveoli	**14**
–	Notogastral setae *c* short, present	**15**
14	Notogastral setae smooth; body length: 468	***Lasiobelba (Lasiobelba) hespiridiana* (Pérez-Íñigo, 1986)**. Distribution: Mediterranean
–	Notogastral setae barbed; body size: 787–825 × 495–539	***Lasiobelba (Lasiobelba) rubida* (Wallwork, 1977)**. Distribution: Santa Helena Islands
15	Interlamellar setae shorter than lamellar setae; body size: 413–600 × 228–336	***Lasiobelba (Lasiobelba) pori* (Vasiliu & Ivan, 1995)** (= *Lasiobelba arabica* Mahunka, 2000, = *Lasiobelba (Lasiobelba) neonominata* Subías, 2004 (see Kok 1967)^3^. Distribution: Ethiopian and Palaearctic regions, Hawai
–	Interlamellar setae longer or similar in length to lamellar setae	16
16	Rostrum tripartite; interbothridial region with three pairs of muscle sigilla; body size: 500–540 × 253	***Lasiobelba (Lasiobelba) decui* (Vasiliu & Ivan, 1995)**. Distribution: Israel
–	Rostrum rounded; interbothridial region with two pairs of muscle sigilla	**17**
17	Bothridial setae with numerous barbs; notogastral setae *p*_3_ longer than *p*_1_ and *p*_2_; body size: 400–530 × 215–280	***Lasiobelba (Lasiobelba) arcidiaconoae* (Bernini, 1973)**. Distribution: Mediterranean, India
–	Bothridial setae with several short barbs; notogastral setae *p*_3_ similar in length to *p*_1_ and *p*_2_; body size: 313 × 233	***Lasiobelba (Lasiobelba) kuehnelti* (Csiszár, 1961)**. Distribution: Oriental, Australian and Ethiopian regions
18	Heterotrichy of dorsal notogastral setae well developed, *la* and *lm* considerably longer than *lp*	**19**
–	Heterotrichy of dorsal notogastral setae absent or weakly expressed, *la* and *lm* not longer than *lp*	**20**
19	Notogastral setae *la* long, reaching the insertions of *lp*; lamellar setae longer than rostral setae; body size: 456 × 216	***Lasiobelba (Antennoppia) quadrisetosa* Subías, 1989** – see Subías and Balogh 1989 (see also Mahunka 2001). Distribution: Greece
–	Notogastral setae *la* of medium size, not reaching the insertions of *lp*; lamellar setae shorter than rostral setae; body size: 498–547 × 298–332	***Lasiobelba (Antennoppia) chistyakovi* Ermilov & Kalúz, 2012**. Distribution: Ecuador
20	Dorsal notogastral setae long, *lm* reaching the insertions of *lp*	**21**
–	Dorsal notogastral setae of medium size or short, *lm* not reaching the insertions of *lp*	**29**
21	Notogastral setae *la*, *lm*, *lp* longer or similar in length to bothridial setae	**22**
–	Notogastral setae *la*, *lm*, *lp* shorter than bothridial setae	**25**
22	Apodemes IV absent; adanal lyrifissures located diagonally to anal aperture; body size: 745 × 510	***Lasiobelba (Antennoppia) insignis* Balogh, 1970**. Distribution: New Guinea
–	Apodemes IV present; adanal lyrifissures located longitudinally to anal aperture	**23**
23	Bothridial setae smooth; body size: 590 × 330	***Lasiobelba (Antennoppia) subnitida* (Sellnick, 1924)**. Distribution: Brazil
–	Bothridial setae barbed	**24**
24	Exobothridial setae longer than rostral setae; bothridial setae similar in length to interlamellar setae; body size: 996–1278 × 697–830	***Lasiobelba (Antennoppia) nepalica* sp. n.** Distribution: Nepal
–	Exobothridial setae shorter than rostral setae; bothridial setae longer than interlamellar setae; body size: 820–861 × 541–574	***Lasiobelba (Antennoppia) granulata* (Mahunka, 1986)**. Distribution: Tanzania
25	Rostrum pointed; body size: 715–800 × 448–486	***Lasiobelba (Antennoppia) major* (Mahunka, 1983)**, see Mahunka 1983a. Distribution: Tanzania
–	Rostrum rounded	26
26	Interlamellar setae represented by alveoli; body size: 590–623 × 232–250	***Lasiobelba (Antennoppia) trichoseta* (Mahunka, 1983)**, see Mahunka 1983b. Distribution: Tanzania
–	Interlamellar setae well developed	27
27	Dorsal notogastral setae inserted in four subparallel rows; interbothridial region with one pair of triangular ridges; body size: 810–1180 × 510–526	***Lasiobelba (Antennoppia) yoshii* (Mahunka, 1987)**. Distribution: Borneo
–	Dorsal notogastral setae inserted in two parallel rows; interbothridial region without triangular ridges	28
28	Interlamellar setae longer than lamellar setae; interbothridial region with three pairs of muscle sigilla; body size: 740 × 450	***Lasiobelba (Antennoppia) capilligera* (Berlese, 1916)** (see also Mahunka 1991). Distribution: Ethiopian region
–	Interlamellar setae slightly shorter than lamellar setae; interbothridial region without muscle sigilla; body size: 555–652 × 314–367	***Lasiobelba (Antennoppia) minor* (Mahunka, 1983)**, see Mahunka 1983a. Distribution: Tanzania
29	Notogastral setae *c* represented by alveoli; rostrum with protruding ledge; body size: 565–605 × 315–335	***Lasiobelba (Antennoppia) ultraciliata* (Jacot, 1934)**. Distribution: Australian region
–	Notogastral setae *c* short, present; rostrum rounded, without protruding ledge	**30**
30	Interlamellar setae similar in length to lamellar setae; exobothridial setae similar in length to rostral setae, respectively; body size: 347 × 185	***Lasiobelba (Antennoppia) heterosa* (Wallwork, 1964)**. Distribution: Ethiopian and Palaearctic regions
–	Interlamellar setae longer than lamellar setae; exobothridial setae shorter than rostral setae; body size: 525–637 × 288–337	***Lasiobelba (Antennoppia) izquierdoae* Arillo, Gil-Martin & Subías, 1994**. Distribution: Canary Islands

## Supplementary Material

XML Treatment for Lasiobelba (Lasiobelba) daamsae

XML Treatment for Lasiobelba (Antennoppia) nepalica

## Footnotes

^1^ Results of the Himalaya Expeditions (1980, 1983) of Dr. Jochen Martens.

^2^ *Lasiobelba (Antennoppia) rigida* was very poorly described by Ewing (1909), therefore we did not include this species in the key.

^3^ *Lasiobelba (Lasiobelba) pori*, *Lasiobelba (Lasiobelba) arabica* and *Lasiobelba (Lasiobelba) neonominata* Subías, 2004 (= *Oppia yodai africana* Kok, 1967 “nom. praeoc.” by Evans, 1953) morphologically are very similar among themselves. We have found any significant morphological differences. Hence, we suggest that *Lasiobelba (Lasiobelba) arabica* and *Lasiobelba (Lasiobelba) neonominata* Subías, 2004 are junior subjective synonyms of *Lasiobelba (Lasiobelba) pori*.

## References

[B1] Arillo A Gil-Martin J Subías LS (1994) Oribatidos del “M.S.S.” de las Islas Canarias. Poroscheloribatinae subfam. n. (Acari, Oribatida). Mém. Biospéol. 21: 1–6

[B2] Aoki J (1959) Die moosmilben (Oribatei) aus Südjapan. Bulletin of the Biogeographical Society of Japan 21(1): 1–22

[B3] Balogh J (1970) New oribatids (Acari) from New Guinea. II. Acta Zoologica Academiae Scientiarum Hungaricae 16(3–4): 291–344

[B4] Balogh J (1983) A partial revision of the Oppiidae Grandjean, 1954 (Acari: Oribatei). Acta Zoologica Academiae Scientiarum Hungaricae 29(1–3): 1–79

[B5] Balogh J Mahunka S (1967) New oribatids (Acari) from Vietnam. Acta Zoologica Academiae Scientiarum Hungaricae 13(1–2): 39–74

[B6] Berlese A (1916) Centuria terza di Acari nuovi. Redia 12: 289–338

[B7] Bernini F (1973) Notulae oribatologicae VII. Gli Oribatei (Acarida) dell’isolotto di Basiluzzo (Isole Eolie). Lav. Del. Soc. Ital. Biogeogr., Nuov. Ser. 3: 355–480

[B8] Csiszár MJ (1961) New oribatids from Indonesian soils (Acari). Acta Zoologica Academiae Scientiarum Hungaricae 7(3–4): 345–366

[B9] Ermilov SG Kalúz S (2012) Two new species of Oppiidae (Acari: Oribatida) from Ecuador. International Journal of Acarology 38(6): 521–527. doi: 10.1080/01647954.2012.687499

[B10] Ermilov SG Kalúz S Martens S (2014) Additions to the Indian oribatid mite fauna, with description of a new species of the genus *Niphocepheus* (Acari, Oribatida). Systematic & Applied Acarology 19(1): 58–66. doi: 10.11158/saa.19.1.4

[B11] Ermilov SG Martens J (2014) Additions to the Nepalese oribatid mite fauna, with description of two new species (Acari, Oribatida). International Journal of Acarology 40(2): 123–132. doi: 10.1080/01647954.2013.870227

[B12] Ermilov SG Martens J Tolstikov AV (2013) New species of oribatid mites of the genera *Lepidozetes* and *Scutozetes* (Acari, Oribatida, Tegoribatidae) from Nepal. ZooKeys 339: 55–65. doi: 10.3897/zookeys.339.6199 24146586 10.3897/zookeys.339.6199 PMC3800828

[B13] Ewing HE (1909) New American Oribatoidea. Journal of the New York Entomological Society 17(3): 116–136

[B14] Jacot AP (1934) Some Hawaiian Oribatoidea (Acarina). Bernice P. Bishop Museum bulletin, Honolulu, 121: 1–99

[B15] Kok DJ (1967) Studies on some South African Oppiidae Grandjean, 1953 (Acarina: Oribatei). The Journal of the Entomological Society of Southern Africa 30(1): 40–74

[B16] Mahunka S (1983a) Oribatids from the eastern part of the Ethiopian region. II. Acta Zoologica Academiae Scientiarum Hungaricae 29(1–3): 151–180

[B17] Mahunka S (1983b) Oribatids from the Eastern Part of the Ethiopian Region (Acari) III. Acta Zoologica Academiae Scientiarum Hungaricae 29(4): 397–440

[B18] Mahunka S (1985) Description and redescription of Ethiopian oribatids (Acari, Oribatida), II. Annales Historico-Naturales Musei Nationalis Hungarici 77: 233–249

[B19] Mahunka S (1986) Oribatids from Africa (Acari: Oribatida) III. Folia Entomologica Hungarica 47(1–2): 41–76

[B20] Mahunka S (1987) Neue und interessante Milben aus dem Genfer Museum LV. Oribatids from Sabah (East Malaysia) I (Acari: Oribatida). Archives des Sciences 40(3): 292–305

[B21] Mahunka S (1991) Notes, additions and redescriptions of the oribatid species of Berlese (Acari). Acta Zoologica Academiae Scientiarum Hungaricae 37(1–2): 27–58

[B22] Mahunka S (1997) Oribatids from Madagascar III (Acari: Oribatida) (Acarologica Genavensia LXXXIII). Revue suisse de zoologie 104(1): 115–170

[B23] Mahunka S (2000) Some oribatid mites from Yemen (Acari: Oribatida) (Acarologica Genavensia LXXXVIII). Annales Historico-Naturales Musei Nationalis Hungarici 92: 325–346

[B24] Mahunka S (2001) Cave-dwelling oribatid mites from Greece (Acari: Oribatida) (Neue und interessante Milben aus dem Genfer Museum XLIX). Revue suisse de zoologie 108(1): 165–188

[B25] Mahunka S Mahunka-Papp L (1995) The oribatid species described by Berlese (Acari). Hungarian Natural History Museum, Budapest, 325 pp

[B26] Norton RA Behan-Pelletier VM (2009) Oribatida. Chapter 15. In: Krantz GW Walter DE (Eds) A Manual of Acarology. Texas Tech University Press, Lubbock, 430–564

[B27] Ohkubo N (2001) A revision of Oppiidae and its allies (Acarina: Oribatida) of Japan 1. Genus *Lasiobelba*. Journal of the Acarological Society of Japan 10(2): 97–109. doi: 10.2300/acari.10.97

[B28] Pérez-Iñigo C (1986) Contribución al conocimiento de los oribátidos (Acari, Oribatei) de la Gomera (Islas Canarias). Eos 62: 187–208

[B29] Sanyal AK (1992) Oribatid Mites (Acari). In: Ghosh AK (Ed) Fauna of West Bengal. Part 3 (Arachnida and Acari) Zoological Survey of India, 213–356

[B30] Sellnick M (1924) Einige neue südamerikanische Damaeosoma Arten. (Acar. Oribat.). Beitr. Tierk. 1: 85–89

[B31] Subías LS (2004) Listado sistemático, sinonímico y biogeográfico de los ácaros oribátidos (Acariformes: Oribatida) del mundo (excepto fósiles). Graellsia 60 (número extraordinario): 3–305 [Online version accessed in February 2014: 577 pp.]

[B32] Subías LS Balogh P (1989) Identification keys to the genera of Oppiidae Grandjean, 1951 (Acari: Oribatei). Acta Zoologica Academiae Scientiarum Hungaricae 35(3–4): 355–412

[B33] Vasiliu N Ivan O (1995) Oribatid mites from Israel. In: Soil fauna of Israel, Editura Academiei Romane, Bucuresti, 69–86

[B34] Vasiliu NA Ivan O (2011) New oppiid species (Acari, Oribatida, Oppiidae) from Romanian caves. Trav. l'Inst. Spéol. “Émile Racovitza” 50: 3–14

[B35] Wallwork JA (1964) Some Oribatei (Acari: Cryptostigmata) from Tchad (1st. series). Rev. Zool. Bot. Afr. 70(3–4): 353–385

[B36] Wallwork JA (1977) Acarina. Cryptostigmata. In: La faune terrestre de L’île de Sainte-Hélène (4eme partie).Mus. Roy. Afr. Centr., Terv., Belg. Ann., Ser. 8, Sci. Zool., 220: 189–257

[B37] Wang H (1993) Three new species of oppiid mites from China (Oribatida: Oppiidae). Acta Arachnologica Sinica 2(2): 97–103

[B38] Wen Z (1999) A new species oribatid mite of the genus *Lasiobelba* from China (Acari: Oribatida: Oppiidae). Acta Zootaxonomica Sinica 24(1): 46–48

